# The effects of crisis plans for patients with psychotic and bipolar disorders: a randomised controlled trial

**DOI:** 10.1186/1471-244X-9-41

**Published:** 2009-07-09

**Authors:** A Ruchlewska, CL Mulder, R Smulders, BJ Roosenschoon, G Koopmans, A Wierdsma

**Affiliations:** 1 Erasmus MC, University Medical Center, Department of Psychiatry, Rotterdam, The Netherlands; 2 Erasmus MC, University Medical Center, Department of Psychiatry, Bavo-Europoort Mental Health Care, Municipal Health Center, Rotterdam, The Netherlands; 3 Cliëntenbond/VOICE, Utrecht, The Netherlands; 4 Bavo-Europoort Mental Health Care, Department of Research and Development, Rotterdam, The Netherlands; 5 Erasmus University Center, Department of Health Policy and Management, Rotterdam, The Netherlands

## Abstract

**Background:**

Crises and (involuntary) admissions have a strong impact on patients and their caregivers. In some countries, including the Netherlands, the number of crises and (involuntary) admissions have increased in the last years. There is also a lack of effective interventions to prevent their occurrence. Previous research has shown that a form of psychiatric advance statement – joint crisis plan – may prevent involuntary admissions, but another study showed no significant results for another form. The question remains which form of psychiatric advance statement may help to prevent crisis situations. This study examines the effects of two other psychiatric advance statements. The first is created by the patient with help from a patient's advocate (Patient Advocate Crisis Plan: PACP) and the second with the help of a clinician only (Clinician facilitated Crisis Plan: CCP). We investigate whether patients with a PACP or CCP show fewer emergency visits and (involuntary) admissions as compared to patients without a psychiatric advance statement. Furthermore, this study seeks to identify possible mechanisms responsible for the effects of a PACP or a CCP.

**Methods/Design:**

This study is a randomised controlled trial with two intervention groups and one control condition. Both interventions consist of a crisis plan, facilitated through the patient's advocate or the clinician respectively.

Outpatients with psychotic or bipolar disorders, who experienced at least one psychiatric crisis during the previous two years, are randomly allocated to one of the three groups. Primary outcomes are the number of emergency (after hour) visits, (involuntary) admissions and the length of stay in hospital. Secondary outcomes include psychosocial functioning and treatment satisfaction. The possible mediator variables of the effects of the crisis plans are investigated by assessing the patient's involvement in the creation of the crisis plan, working alliance, insight into illness, recovery style, social support, locus of control, service engagement and coping with crises situations. The interviews take place before randomisation, nine month later and finally eighteen months after randomisation.

**Discussion:**

This study examines the effects of two types of crisis plans. In addition, the results offer an understanding of the way these advance statements work and whether it is more effective to include a patients' advocate in the process of creating a psychiatric advance statement. These statements may be an intervention to prevent crises and the use of compulsion in mental health care. The strength and limitations of this study are discussed.

**Trial registration:**

Current Controlled Trails NTR1166.

## Background

Crises and (involuntary) admissions have a strong impact on patients and their relatives [1]. Approximately 50% of patients experience involuntary admission as traumatic [2]. Over the years 2000–2003 the number of outpatient emergency service visits in the Netherlands has increased 106%, the number of admissions 162% and involuntary admissions 17%. This increase continued after 2003 [3].

Possible explanations for the aforementioned increases include a shift from inpatient to outpatient services, a tendency to intervene earlier in the crisis situation and to remove homeless people from the street [3]. Another explanation is a lack of suitable outpatient services and early recognition of future crisis situations [4]. Advance statements such as crisis plans are rarely used in metal health practices in the Netherlands. In the UK, according to an unpublished report of Nagaiah and Szmukler (2007) [5], crisis plans belonging to the treatment plans are seldom described, or are very brief and rarely contain good quality information.

There are different kinds of advance statements. The context, such as the involvement of a mental health provider, independent facilitation, or a legislative status defines the statement type. An "Advance directives" is a legally binding document which describes the preferences for and refusals of treatment in advance. An "Advance agreement" is a plan that is jointly agreed upon between patient and mental health provider, for instance a joint crisis plan [5]. Some advance statements are created independently from the mental health provider, such as the so-called 'crisis card'. These are often created with the help of a self-advocacy group.

The effects of advance statements to prevent crises and (involuntary) admissions have scarcely been studied [6]. Two studies examined the effects of two different advance statements. In the first study the so-called joint crisis plan was developed by the patient and his or her outpatient treatment team. The process was facilitated by a mental health professional who was not a member of the treatment team. In the group of patients with whom the 'joint crisis plan' was developed, significantly fewer patients were compulsorily admitted as compared to the control group without such a plan: 13 and 27%, respectively [7]. The second study didn't find any significant effect of a different statement [8]. The intervention consisted of a 'booklet' containing seven statements about future treatment preferences. The patient wrote his or her preferences independently from the outpatient mental health team during involuntary inpatient stay. The advance directives were kept in the patient's records. The explanation for the lack of a result may be that the outpatient clinicians were unaware of the existence of the booklets after the patient's discharge from the hospital. The positive results in the 'Joint Crisis Plan' study suggest that the involvement of an independent facilitator and the outpatient mental health team are essential for preventing involuntary admissions.

Little is known about the mechanisms that cause the possible effects of advance statements. In a study on 'psychiatric advance directives' [9], people who received help to complete the document showed a significantly greater improvement in their working alliance with clinicians and were more satisfied with their treatment than patients in the control group. The process of developing an advance statement may influence coping style and one's insight into illness. Advance statements can enhance treatment self-efficacy and help identifying early signs of a crisis, both by the patients and their clinicians. These mechanisms might empower patients and lead to more treatment adherence [10,11].

In the Netherlands, different forms of advance statements exist. The so-called 'crisis plan' was developed by the Amsterdam Patient and Consumer Advocacy Group in 1999, and can be described as an instruction for mental health emergencies. In this crisis plan two aspects are addressed: crisis prevention and provisioning of practical information for future psychiatric emergency care. The practical information is summarized on a small card, the 'crisis card', which the user carries with him or her at all times. The crisis plan is developed independently from the mental health provider with the help of a patient's advocate; the clinician signs the final document afterwards. According to the advocacy groups, the facilitation by a patient's advocate is an important contribution to the effectiveness of the plan, since a power imbalance occurs between patient and clinician when the crisis plan is created together with the clinician only. The crisis plan may end up being in the interest of the professional instead of the patient's concerns. Involving a patient's advocate may help the patient to better express his wishes in times of crisis. However, questions remain about the effectiveness and practicalities of involving a patient's advocate in the process. It may be equally effective to develop a crisis plan together with the clinician, without the facilitation of a patient's advocate.

### In summary

The numbers of emergency visits and (involuntary) admissions have increased in the Netherlands. Effective interventions are required to prevent a further increase. Advance statements such as crisis plans may be an effective way to prevent emergency visits and (involuntary) admissions. This study examines whether a crisis plan, facilitated respectively through the patient's advocate or the clinician, can reduce the number of emergency visits and (involuntary) admissions. Furthermore, this study seeks to identify possible mediating mechanisms for the effects of these two forms of crisis plans.

### Research aims

This study has three aims. Firstly, to investigate whether there is a differential effect of a crisis plan facilitated through the patient's advocate, or through the clinician, on the number of psychiatric emergency visits and (involuntary) admissions as compared to a control group without a crisis plan. Secondly, to investigate the differential effects of the two different crisis plans on the patient's locus of control in a time of crisis and in social and psychological functioning. The third aim is to identify the mediating mechanisms responsible for the (possible) effects of the crisis plans, including the quality of the therapeutic alliance with the clinician, the patient's recovery style, social support, therapy adherence, self-efficacy and insight into illness.

### Hypotheses

**Figure 1:**
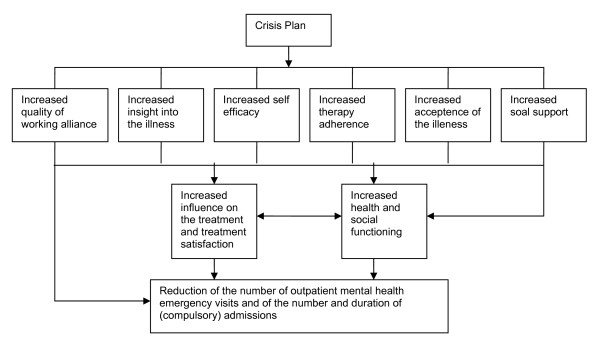
**Figure of the mediator variables of the effects of the crisis plan**.

Regarding the first aim, it is hypothesized that both the crisis plan facilitated through the patient's advocate and by the clinician can reduce the number of psychiatric emergency visits and (involuntary) admissions as compared to the control group without a crisis plan. In addition, we expect a greater effect when the crisis plan is facilitated through the patient's advocate as compared to the crisis plan developed with the clinician only. Furthermore, we expect greater effects on the patient's satisfaction with treatment and psychological functioning when the crisis plan is facilitated through the patient's advocate as compared to the crisis plan developed with the clinician only. Another aim of this study is to investigate the mediating mechanisms of the crisis plan. The expectation is that after creating the crisis plan, patients will show improvements in working alliance, insight into illness, self-efficacy, therapy adherence, acceptance of the illness and social support (see figure 1).

## Methods/Design

The trial is funded by the Dutch organization for health research and development (ZonMw) and the mental health care organisation Bavo Europoort in Rotterdam, the Netherlands.

### Design

This is a randomised controlled trial using two intervention groups and one control group. Group one consists of patients who create a crisis plan with a patient's advocate. The patients in the second group create a crisis plan with their clinician only. The third group is the control group in which the patients do not create a crisis plan. The main outcome measures are the number of the mental health emergency visits, the number of (involuntary) admissions and the length of stay in hospital.

### Participants/Setting

Participants in the study are adult outpatients, between 18 and 65 years of age, with a psychotic or bipolar disorder, and who are at risk of psychiatric crises. Participants are recruited from eighteen community mental health teams in three mental health institutions in Rotterdam, the Netherlands. These teams are located throughout the city centre, the northern, eastern and southern part of Rotterdam and its vicinity.

Inclusion criteria are: having a diagnosis of a psychotic or bipolar disorder, treatment on an outpatient basis and having had at least one crisis contact with mental health services or (compulsory) admission during the previous two years.

Exclusion criteria are: having a somatic disease causing a psychotic disorder, the inability to give informed consent because of mental incapacity, insufficient command of the Dutch language, and already having a 'relapse prevention plan' or a 'crisis plan'.

### Recruitment/procedure

Candidate participants are selected from the clinicians' caseloads by the clinician and the researcher. The selected patients receive an information letter about the study from their clinicians, who request the patient's permission to be contacted by an independent researcher. The researcher explains the research goals and randomisation procedure. After providing written informed consent, the baseline interview follows.

**Figure 2:**
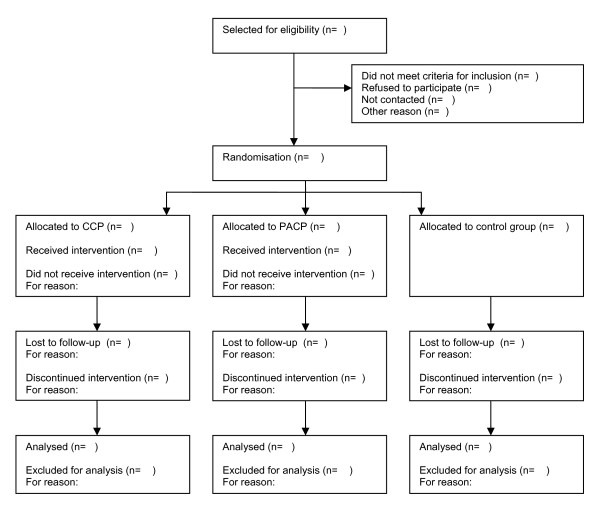
**Participant flowchart**. PACP: Patient's Advocate facilitated Crisis Plan, CCP: Clinician facilitated Crisis Plan.

The second interview with the patient is scheduled nine months later, and the last interview eighteen months after the baseline measurement. Figure 2 represents a participants' flowchart.

### Definition of the intervention

The research intervention includes two forms of crisis plans. The first type of psychiatric advance statement is created by the patient with the help of a patient's advocate (Patient Advocate Crisis Plan: PACP) and the second with the help of a clinician only (Clinician facilitated Crisis Plan: CCP). Both crisis plans describe how to recognize early signs of a crisis and how to provide adequate help. The plans are summarized on a small card the size of a credit card and folded into a plastic wallet that the user carries at all times. The card contains practical information to be used in times of crisis, for example who must be called, or what to do with pets.

The CCP is an advance agreement because the clinician and the patient formulate the content of the crisis plan together. The plan is based upon the principles of the 'shared decision making model' [12]. The PACP is a type of advance statement. In this case the clinician is less involved in the process of formulating the plan and therefore the PACP represents the "autonomy model" [12].

Naturally, crisis plans are constructed on a purely voluntary basis. It can only be formulated if both clinician and patient are willing to cooperate in this process (CCP), or when the patient desires to formulate it with his or her advocate (PACP). The plan is not legally binding, because a clinician may deviate from the content of the plan in times of crises if strictly necessary for the treatment of the patient.

### Chosen type of advance statement

Henderson and colleagues have made a typology of advance statements [5]. Although advance statements in our study (PACP and CCP) show much similarity with advance statements described by Henderson [5], they differ from the described types in certain features. One important difference is the facilitation through a patient's advocate. The patient's advocate formulates the plan with input from the clinician. The two participating patient's advocates in our study are experienced social workers. One of them is a consumer peer specialist. The CCP does not use a patient's advocate to help make the plan. Both the PACP and CCP are disseminated in the mental health administration system.

### Intervention procedure

Before the start of the study, all participating community mental health teams were informed about it during a two hour team meeting with the members of the advocacy group and the researcher.

#### Patient Advocate Crisis Plan: PACP

The procedure in the PACP group is as follows. After the patient has been randomized to this condition, the patient's advocate makes an appointment. During the first meeting, the advocate discusses the procedure with the patient and collects information for the crisis plan. Crises-precipitating factors are discussed and strategies for preventing crises are developed. After this meeting, the advocate prepares the first concept of the plan. The patient, supported by the advocate, negotiates with his or her clinician about what to do when the first signs of a crisis develop and what his or her wishes are about what to do in times of crisis. When the plan is ready, it is signed by the patient's psychiatrist, the clinician (most likely a psychiatric nurse) and other people (e.g. the partner, friends or family) involved in the crisis plan. The final step is to summarize the plan on a crisis card, which is then handed to the patient. The content of the crisis plan is to be evaluated annually or more frequently if necessary. The time period needed to complete the plan and the number of contacts with the patient's advocate and the clinician will be registered during the study.

#### Clinician facilitated Crisis Plan: CCP

After randomisation to the CCP condition, the clinician is provided with the CCP protocol and the researcher explains the structure of the intervention in more detail. As in the PACP condition, crises-precipitating factors are discussed and strategies are developed for preventing them. The patient and his or her clinician formulate the content of the crisis plan together. The procedure contains several stages: the preparation and formulation of the crisis plan, an informed discussion, and the collection of signatures of everyone involved in the development process (e.g. the partner, friends or family). The content of the crisis plan is to be evaluated annually or more frequently if necessary. The final step is to summarize the plan on a crisis card, which is then handed to the patient. The time period needed to complete the CCP and the number of contacts with the clinician for making the CCP will be registered during the study.

#### Structured monitoring

Every three weeks the researcher inquires with the patient's advocate (in de PACP condition) or the clinician (in the CCP condition) regarding the progress and possible problems involved in making the crisis plan. Supervision meetings are organized for the clinicians in the CCP group. During these meetings clinicians have an informed discussion and learn from each other's experience with crisis plans.

A checklist is developed to examine the quality of the finished crisis plans. This checklist refers to the 10 items of the crisis plan and is scored from 0 'vague/no description' to 4 'complete/concrete description'. Two independent research assistants assess the quality of the plans using this checklist.

#### Dissemination method

All crisis plans are included in the patients' records and in the electronic records of all emergency psychiatric services that the patient may come into contact with during a crisis (i.e. crisis centre, crisis teams, and admissions wards).

### Instruments

#### Baseline variables

Demographic variables, psychiatric history and diagnoses are collected from the patient's records.

### Primary outcome measure

Primary outcome measures are the number of the crisis contacts with the clinician or after-hours emergency services, the number of (involuntary) admissions and the length of stay in hospital. The data are collected from the patient administration system and the emergency services' electronic system.

### Secondary outcome measures

Secondary outcome measures include health and social functioning, the patient's influence on crises situations and treatment satisfaction.

#### Health and social functioning

This is assessed by an independent interviewer using the Dutch version of the Health of the Nation Outcome Scales [13,14]. The HoNOS form is completed by the researcher after a structured interview to quantify the health and social problems during the previous two weeks. Twelve items refer to behavioural problems, impairment, symptoms and social (dis)functioning. Three HoNOS-addendum items refer to manic symptoms, treatment motivation and compliance with medication. The items are rated from 0 'no problem' to 4 'severe to very severe problem'.

#### Evaluation of Crisis Plan (ECP)

The patient's opinion regarding the quality of the crisis plan's creation process will be assessed using a newly developed 13-item self-report *Evaluation of Crisis Plan *questionnaire. Specifically, the patient is asked whether he or she feels that the crisis plan reflects his or her wishes about what to do during a crisis. The items are rated on a 5 point scale, from 0 'no, I strongly disagree' to 4 'yes, I strongly agree'.

#### Mental Health Care Thermometer (MHC-T)

Treatment satisfaction is measured according to the *Mental Health Care Thermometer *[15,16]. The 16 items on this scale consist of "yes" or "no" categories that refer to the patient's satisfaction regarding the treatment information received, the patient's involvement in the treatment planning, the patient's impression of the clinician and of the treatment quality.

### Mediator variables

Possible mediator variables include working alliance, insight into illness, recovery style, social support, locus of control, service engagement and coping with (advance) crises situations.

#### Working alliance

The quality of the working alliance is measured by the Dutch version of the *Working Alliance Inventory *(WAI) [17,18]. This questionnaire is measured from both the patient's and the clinician's perspective. The 33 items are rated on a 5 point scale, from 0 'no, I strongly disagree' to 4 'yes, I strongly agree'. The reliability of the *scale *is adequate.

#### Illness insight (PI)

This self-report scale measures the insight into psychosis [19]. There are eight statements to which the participant may respond in one of three ways: agree, disagree and unsure. Three subscales refer to the relabeling of symptoms, awareness of illness and the perceived need for treatment. The English version of the scale has strong psychometric properties.

#### Coping with crisis (CC)

The patient's ability to cope with crisis situations (self efficacy) is measured with a newly developed 21-item self-report questionnaire. Answers are rated on a 5 point scale from 1 'strongly disagree' to 4 'strongly agree'. The items refer to five dimensions: 1) control of one's own treatment, 2) how to prevent a crisis, 3) how to recognise a crisis, 4) knowing what to do in case of an advance crisis and 5) knowing what to do in a crisis situation.

#### Locus of control (MASTERY)

The patient's personal feeling of control over the forces that impact their own life is measured with a 7-item scale [20]. Each item is a statement regarding the respondent's perception of self. Four responses are rated from 1 'strongly disagree' to 4 'strongly agree'. The psychometric properties of this scale are adequate.

#### Service engagement (SES)

The Service Engagement Scale is used from the clinician's perspective [21,22]. The 14 items are rated on a 4 point scale, from 0 'not at all or rarely' to 3 'most of the time'. The three subscales refer to availability, collaboration, help seeking and treatment adherence. The English version of the scale has good psychometric properties.

#### Recovery style (RSQ)

The *Recovery Style Questionnaire *measures the extent to which the patient accepts or denies his or her illness [23] (Table 1). The 39 items have 'agree' and 'disagree' answer categories.

**Table 1:** Instruments at three research contacts

	**M1 Baseline**	**M2 9 months**	**M3 18 months**
**WAI**	X	X	X
**PI**	X	X	X
**CC**	X	X	X
**MHC-T**	X	X	X
**ECP**		X	
**RSQ**	X	X	X
**MASERY**	X	X	X
**SERVES**	X	X	X
**ASR**	X	X	X
**HoNOS**	X	X	X

WAI: *Working Alliance Inventory*, PI: *Psychosis Insight*, CC: *Coping with Crisis*, MHC-T: *Mental Health Care Thermometer*, ECP: *Evaluation of Crisis Plan*, RSQ: *Recovery Style Questionnaire*, MASTERY: *Control of one's life events*, SERVES: *Service Engagement Scale*, ASR: *Adult Social Report*, HoNOS: *Health of the Nation Outcome Scale*.

The English version of the *Recovery Style Questionnaire *has an adequate reliability.

#### Social support (ASR)

Social support is measured with the *Adult Social Report *scale [24,25]. This self-report scale includes fourteen items. Each item is rated on a five point scale from 'no help at all' to 'very much help'. The scale's reliability is good.

### Randomisation

Randomisation is stratified by team. To ensure the even distribution of the patient groups within each team, envelopes with 12 lots per team are used. After completing the baseline interview, the interviewer requests allocation by email. The principal investigator allocates participants into one of the three groups (PACP, CCP and control group).

### Power

The difference for the primary outcome variable between the intervention groups and the control group is based on a power of 0.90 and an alpha of 0.05. To detect an effect size of 0.6, each condition requires a minimum of 50 subjects. We have decided to use 80 subjects in each group to make up for those that we anticipate will be lost in the follow-ups.

### Statistical analyses

Analysis will be performed according to the *intention-to-treat *principle. Group difference will be investigated by chi-square tests and an(c)ova.

A patient is a study completer when he or she has completed all three interviews. After the intention to treat analyses, we will also analyse the effects in those patients who have completed the crisis plan. The missing data of secondary and mediating variables will be replaced by the data of the last available measurement using the principle of *last observation carried forward*.

### Ethical principles

The study protocol, information brochure and informed consent form were approved by the Dutch Union of Medical-Ethic Trail Committees for mental health organizations (registration number 7.109, CCMO-nr NL 16818.097.07).

The effects of a crisis plan are unknown at this moment and therefore we think it is justified to allocate the participants randomly over the three conditions.

The clinician informs the patient about the research. After permission is granted, the interviewer informs the patient of the research aims and randomisation method and asks for his or her written informed consent. The patient is free to refuse participation at any time during the research period, without having to disclose any reason why.

Participants allocated in the control group receive care according to standard practice, without the creation of a crisis plan. In case patients in the control group wish to create a crisis plan, this will be honoured at any time during the research period.

The collected patient data are treated according to the Medical Confidentiality Rules, and are kept in locked files cabinets. Access is limited to members of the research group and the medical ethical committee.

## Discussion

The central research question in this study is whether either of the two crisis plan types can reduce the use of psychiatric emergency services, as well as the number and duration of (involuntary) admissions. The secondary research question is whether the intervention improves psycho-social functioning. The identification of the possible intervention mediating mechanisms offers a tool for use in the development of future preventive interventions. The comparison between the two crisis plan types provides insight into the question whether a crisis plan facilitated through the patient's advocate is more effective than a crisis plan facilitated through the clinician only. The study has several limitations and strengths.

### Limitations

Firstly, no structured diagnostic interview is used to confirm the DSM-IV diagnosis. We decided to use the clinical diagnosis as derived from the medical records because of the extensive nature of the interview, and because a structured diagnostic interview-derived DSM-IV diagnosis is of limited importance in the present study. The second limitation is the possible recruitment bias. Because of the ethical consideration the clinician is the first person who informs the patient of the study. Some clinicians may have preferences for some patients to participate in the study. There is some risk that it will not be possible to generalise the results based on the expected response of about forty percent of the participants [7,8]. People who don't want to participate may have experienced a compulsory admission in the past and feel demoralized and disempowered.

### Strengths

Important strengths are the clinical relevance and design of this study. Although crisis plans are formally part of the treatment plans, in practice clinicians rarely use advance statements. The structure and supervision provided by this study will help participating clinicians to switch their working method into a more structured and preventive approach.

The participants are not screened for their ability to make a crisis plan and therefore represent a more general population of patients with psychotic and bipolar disorders than a selected group of patients. Besides that, the multisite character of this study may also increase the generalization of the results. Internal validity is protected by the structured protocol monitoring and supervision of the clinicians.

This study is jointly developed and conducted with the patient's advocacy group and, to our knowledge, is therefore the first randomised controlled trial which examines such an intervention.

## Competing interests

The authors declare that they have no competing interests.

## Authors' contributions

AR wrote this manuscript and is the study's principal investigator. CLM designed and leads the project and helped to draft the manuscript. BR and RS co-designed and supervise the project. GK is the project's statistical advisor. AW is a health services research specialist. All authors provided comments and read the manuscript.

## Pre-publication history

The pre-publication history for this paper can be accessed here:


